# Being one of us: we-identities and self-categorization theory

**DOI:** 10.1007/s11097-023-09923-0

**Published:** 2023-07-17

**Authors:** Felipe León

**Affiliations:** Department of Thematic Studies: Technology and Social Change, and Centre for Medical Humanities and Bioethics, Linköping University, Campus Valla, TEMA-huset, Rum A:333, s-581 83, Linköping, Sweden

**Keywords:** We-identity, Self-categorization theory, Depersonalization, Close personal relationships, Socially extended mind

## Abstract

One way to theorize about we-identities—the identities that individual subjects have as ‘one of us’—is in terms of the uniformity, interchangeability, and prototypicality of group members. The social-psychological theory of self-categorization epitomizes this approach, which has strongly influenced contemporary phenomenological research on the we. This paper argues that this approach has one important and largely overlooked limitation: the we-identities tied to close personal relationships—exemplified by long-term friendships and romantic partnerships—are based on patterns of interpersonal interaction and integration through which individuals tend to grasp their non-substitutability and complementarity. This limitation suggests that another approach is needed to tackle the we-identities characteristic of close personal relationships. I outline such an approach, by combining resources from classical phenomenology and ongoing research on the socially extended mind.

## Introduction

The sense of belonging to social groups—such as families, romantic partnerships, nation states, political parties, etc.—is a familiar and widespread feature of the human social world. Intuitively, group belongingness plays a central role in shaping the contours of individuals’ identities. One natural way to answer the question ‘who are you?’ is, at least in part, by telling about some of the groups and social categories that one takes oneself to belong to. Psychological research indicates that belongingness is not merely an occasional desire or expectation that people have, but rather a basic and nearly universal psychological need (Baumeister & Leary, 1995; Fiske, 2014, pp. 16–17). Beyond this, it has been recently argued that belongingness is not only a social need, but also a human right (Brownlee, 2020).

The overall goal of this paper is to investigate the sense of belonging by focusing on the notion of we-identity. Although this notion is not new in the philosophical literature (see e.g. Schmid, 2005; Zahavi, 2015a, p. 97), and has appeared sometimes under labels such as “collective identity” (e.g. Mathiesen, 2003), “group identity” (e.g. Young, 1990), and “social identity” (e.g. Appiah, 2014), it is much less explored than the widely investigated notion of collective intentionality (see Schweikard & Schmid, 2020). The situation is somewhat different in the social sciences, where notions such as collective identity, group identity, and social identity have an important trajectory in disciplines like social psychology and sociology (see, for example, Ashmore et al., 2004; Flesher Fominaya, 2010).

In recent years, work in social psychology has started to influence philosophical research about the we informed by research in classical and contemporary phenomenology (Taipale, 2019; Zahavi, 2019; Salice & Miyazono, 2020; Salice & Henriksen, 2015). The present paper is intended as a contribution to this interdisciplinary dialogue. My central aims will be to articulate one important and largely overlooked limitation of the resources that the social-psychological theory of self-categorization (e.g. Turner et al., 1987) can offer for the investigation of we-identities, and to outline one way in which that limitation can be overcome.

The paper is structured as follows. In Section 2, I outline a characterization of we-identities aimed at clearing up some terminological issues. Section 3 presents the main tenets of the theory of self-categorization and explores some of its connections with classical and contemporary phenomenological work on the we. Section 4 argues that self-categorization theory and current proposals informed by it don’t offer suitable resources to explain the we-identities tied to close personal relationships, paradigmatically exemplified by long-standing romantic partnerships and friendships. The reason is that they take a first-person plural perspective to involve a psychological process of “depersonalization” (Turner, 1987, p. 50) or “de-individuation” (Salice & Henriksen, 2021, p. 11; Zahavi, 2019, p. 256), through which subjects accentuate their homogeneity, interchangeability, and adherence to an in-group prototype. While such features may be contingently present in close relationships, I argue that there are good reasons to support the view that the we-identities characteristic of them arise from processes that go in a quite opposite direction from “depersonalization”. In Section 5, I outline one way in which resources from classical phenomenology and ongoing research on the socially extended mind can shed light on the we-identities characteristic of close relationships.

## Characterizing we-identities

The notion of identity relevant in the expression ‘we-identity’ is not primarily identity as re-identification and continuity across time—familiar from philosophical discussions about personal identity—, but rather a more vernacular notion of identity, understood as the set of characteristics that make someone the person she or he is (Schechtman, 1996, p. 2). To a first approximation, we-identities are identities that individual subjects have as ‘one of us’, in virtue of their membership in social groups. Making this description more precise will be the main task of this section. For the moment, it suffices to motivate the question of why would a focus on the notion of we-identity be appropriate, given that there are other notions in the literature—such as social identity, collective identity, and group identity—that seem to target the relevant *explanandum*. The short answer is that the other notions are prone to various misunderstandings, and that this justifies a terminological intervention.

Starting with the notions of group identity and collective identity, both are ambiguous concerning the subject of attribution. On the one hand, one can take it to be the group or collective as a supra-individual unit—on this reading, ‘group identity’ refers to a *group’s* identity (e.g. Gallagher, 2020, p. 219) and ‘collective identity’ to a *collective’s* identity (e.g. Rucht, 1995, p. 10). On the other hand, one can take the subject of attribution to be an individual insofar as he or she is a member of a group or collective (e.g. Bacharach, 2006, p. 69; Sullivan, 2014, p. 1). Apart from this ambiguity, the notion of collective identity tends to have political undertones (Ashmore et al., 2004, p. 81), which might be justified in some contexts (e.g. social movements research (see Flesher Fominaya, 2010) but not in others.

The notion of social identity doesn’t have the same limitations, as it has been typically used for referring to an identity that an individual subject has qua group member, quite independently of whether the group in question has a political character (e.g. Tajfel and Turner, 1986). But it has one noticeable disadvantage. Since there is a wide consensus that sociality pervasively shapes the identities of persons, it isn’t really clear what notion(s) of (non-social or pre-social) identity the concept of social identity should be distinguished from. This limitation of the notion becomes clear in the work of John Turner and his collaborators, whose use of the term *social identity* is not supposed to pick out one aspect of self-identity marked by its social character, as distinguished from other aspects of self-identity that would lack that character (Turner et al., 1987, p. 46).^1^ This is nonetheless a misleading connotation of the term, which has prompted some commentators to drop it altogether (Simon & Klandermans, 2001, p. 320; Ashmore et al., 2004).

In light of the above, the notion of we-identity seeks to avoid shortcomings of other available notions, by emphasizing a central idea that is nonetheless hinted at by some of them. This is the idea that there are aspects of an individual’s self-conception that he or she has qua member of a social group. To that extent, those aspects have a first-person plural character. Were one to ask someone to articulate what it means to have one of those aspects, it would be natural to do so by using the first-person plural or ‘we’ pronoun.

For the purposes of this paper, I will adopt a broad definition of social groups, according to which “x is a social group if and only if x is an entity constituted by and only by people” (Epstein, 2019, p. 4914). This broad definition avoids the risk of “under-generating” social groups and vindicates the idea that “[d]ifferent kinds of social groups have little to unify them aside from their being built of people” (2019, p. 4914).^2^ Moreover, this broad definition circumvents the difficulty of how to adjudicate between narrower definitions of social group that run the risk of being ad hoc or that are guided by more specific theoretical agendas (e.g. Young, 1990, p. 43). Based on this definition, we can start with the task of characterizing the notion of we-identity by considering the following *Membership Condition*^3^:

(i) A we-identity is an identity that an individual subject has in virtue of their membership in a social group.

Since membership in some groups doesn’t require attitudes of a member towards the group in question, this condition is very inclusive (see Zahavi, 2015a, p. 94). For one might be a member of a group of people without knowing about the group’s existence, let alone about one’s membership in it. For example, one might be a member of the group of people who have a certain genetic predisposition, without knowing that such group exists. And even if one knows that the relevant group exists, one might nonetheless ignore that one is a member. Two comments are in order here. The first one is that the idea that membership in some social groups is attitude-independent need not mean that membership should be conceptualised as a brute fact of the world. Perhaps at bottom the very idea of membership in a social group depends (at least potentially) on the recognition from other members of the group in question. I won’t delve into this issue in the present context. The second comment is that there are domains—such as social policy-making—in which it might be useful to operate with a very broad understanding of group membership. Policy makers are sometimes concerned with locating individuals under certain social categories, quite independently of whether people may have any sense of belonging to them. However, given the focus of the present paper, the very broad understanding of a we-identity suggested in (i) will contribute little to an elucidation of the sense of belonging, which involves reference to the experience of being a group member. In other words, even though group membership is a necessary condition for a we-identity, it is not sufficient.

We get a more restricted notion of we-identity by introducing the following *Awareness Condition*:

(ii) A we-identity is an identity that a subject is, at least at times, aware of having.

This condition captures the idea that individual subjects are sometimes aware of their membership in some social groups (Zahavi, 2015a, p. 95). This awareness doesn’t have to be thematic or reflective (see Schmid, 2005), which means that it doesn’t require an explicit focus on the relevant group membership. Moreover, condition (ii) doesn’t require either that awareness of membership is persistent, but only that it can become experientially salient at times and, we may add, under suitable conditions. We-identities have a dynamic and fluid character, since they can become more or less experientially salient in different contexts (see Taipale, 2019, p. 232). Awareness of membership in a social group is not (typically, at least) a persistent feature of a subject’s psychological life. Moreover, such awareness can be grounded in a dispositional belief of membership in the relevant social group.

But being occasionally aware of one’s membership in a social group doesn’t provide yet a sufficiently delimited characterization of a we-identity. The reason is that one might be occasionally aware of one’s membership in a social group, but membership may be attributed or even imposed by others, without one having any say in that attribution. In some cases, group membership is attributed by others in ways that are diminishing and unjust, and a convincing characterization of we-identities should be responsive to this feature of the social world. However, at least part of the reason why such cases seem problematic is that they involve a discrepancy between group memberships attributed by others and self-attributed group memberships. If so, a proper understanding of imposed group memberships will presuppose an understanding of what happens when group membership is self-attributed. To capture this idea, I introduce a further condition, which I call *Endorsement Condition*:

(iii) A we-identity is an identity that a subject endorses or appropriates.

To endorse of a particular identity as member of a social group goes beyond having an occasional awareness of that membership. Endorsement is a matter of assenting to a group membership and incorporating or appropriating it as part of one’s self-conception, however fleetingly that might happen.^4^ At the same time, endorsement can happen in a more or less peripheral manner, because we-identities are aspects of one’s self-conception that tend to be weighted differently—depending on factors such as the place they have in one’s overall evaluative outlook and relevant contextual elements. It seems plausible to assume that the differential evaluation of endorsed group memberships will be reflected in the affective salience that group memberships tend to have (Taipale, 2019, p. 233). This means that the significance of an endorsed group membership will tend to be disclosed in the affective attitudes that the subject has when it comes to group-related issues. Accordingly, I introduce the following condition of *Affective Salience*:

(iv) We-identities vary in terms of their affective salience.

In short, we-identities are identities that subjects care about differentially. It is important to note that affective salience need not be tied to positive sentiments. Perhaps some memberships are not easily embraced, or perhaps there can be many ambivalences about one’s membership in a group, yet that doesn’t mean that such memberships are not affectively salient (quite to the contrary, perhaps).

Summing up, on the present characterization, a we-identity is one aspect of a subject’s self-conception had in virtue of the subject’s membership in a social group. Such membership is something that the subject is, at least at times, aware of having, that he or she endorses, and that will tend to be more or less affectively salient. This characterization, which builds on recent phenomenological work on the first-person plural perspective, aims at capturing the we-identities tied to a wide array of social groups that play a central role in social life. These include nation states, ethnic groups, corporations, groups of colleagues, families, groups of friends, and groups of romantic partners. In spite of their many differences, a common denominator of these (and other) social groups is that they allow for subjects to have we-identities, along the lines presented above. It is an advantage of the present characterization that it can be applied to many sorts of groups, quite independently of any particular group taxonomy that one might be inclined to adopt (see, e.g., Lickel et al., 2001).

## Self-categorization theory, social identity, and the “public person”

How to account for we-identities? One proposal that has gained momentum in contemporary phenomenology is that the we requires that individuals identify with a group by accentuating their perceived uniformity and homogeneity (e.g. Salice and Henriksen, 2015, pp. 158–159; Zahavi, 2019, pp. 255–256). A few considerations about the context and motivation for this proposal are in order here, before turning to the theory of self-categorization. The phenomenological tradition is well known not only for providing ground-breaking analyses of subjectivity, consciousness, and self-consciousness, but also for extensive investigations of the social world. Needless to say, the latter is not a homogenous domain. It encompasses a wide range of phenomena, such as the attribution of mental states to other people, doing things together with others, the sharing of emotional experiences with them, and the sense of belonging to social groups.

Importantly, as this rough description indicates, there is a sense in which the social world is not only about the first-person *singular* perspective of an experiencing subject, but also (and perhaps even primarily) about the first-person *plural* perspective of a plurality of subjects who partake in various shared and collective engagements. In brief, the social world is not only about the ‘I’, but also about the ‘we’. One question that arises here concerns the status of the first-person plural perspective. Given phenomenology’s long-standing interest and emphasis on the distinctive and irreducible character of the first-person *singular* perspective—and assuming that the ‘we’ doesn’t reference any supra-individual experiencing subject—how to account phenomenologically for the first-person *plural* perspective? It is important to emphasize the broad scope of this question. The question is not supposed to focus on one particular sub-domain of the social world in which a particular type or configuration of the first-person plural perspective is discernible. It aims rather at investigating general features of that perspective. In brief, what is at stake is “*the* we” (Zahavi, 2015b, p. 154. My emphasis), and not just *a* (particular type of) we.

This is the theoretical context in which the idea that the we requires that individual subjects accentuate their perceived similarity and homogeneity has started to gain traction. Now, since group processes and the we have been long-standing topics of interest in other disciplines, particularly in the social sciences, it is not very surprising that contemporary phenomenological research—with its strong interdisciplinary inclinations—has appealed to resources from disciplines like social psychology (e.g. Salice and Henriksen, 2015, p. 156; Zahavi, 2019, p. 256). In fact, one main strand of empirical support for the proposal that the we requires an accentuation of perceived similarity and homogeneity has been found in the social-psychological theory of self-categorization (Turner et al., 1987, 1994), to which I now turn.

Self-categorization theory is an established research paradigm in social psychology, which grew out as a generalized form of the social identity theory first put forward by Henri Tajfel (Tajfel, 1978; see Hornsey, 2008). Whereas social identity theory was primarily focused on inter-group behaviour, self-categorization theory is concerned with “how individuals are able to act as a group at all” (Turner et al., 1987, p. 42). A guiding idea of self-categorization theory is that individual subjects categorize themselves at different “levels of self-representation” (Turner et al., 1987, p. 45). Although the theory doesn’t say much about the very notion of self-representation that it operates with, one can take it to mean, roughly, a way in which a subject understands and thinks of herself, and acts accordingly (for discussion, see Salice and Miyazono, 2020).^5^ Crucial in the context of self-categorization theory is the distinction between two types of self-representation, called *personal identity* and *social identity*.^6^*Personal identity* comprises idiosyncratic features that distinguish self from others and define the self as unique (Turner et al., 1987, p. 45). In contrast, *social identity—*the main focus of self-categorization theory—is defined as “those aspects of an individual’s self-concept based upon their social group or category memberships together with their emotional, evaluative and other psychological correlates” (Turner et al., 1987, p. 29).

According to self-categorization theory, instances of these types self-representations tend to be activated and become salient in specific situations, as a function of their accessibility (the degree to which they are poised to being activated) and their fit (the degree to which perceived stimuli match the specifications of a given categorization) (Turner et al., 1987, pp. 44, 55). It should be underlined that *social identity* and *personal identity* are not supposed to encompass a stable set of trait-like features. In fact, one might suppose that, under suitable conditions, the very same feature (such as, suppose, ‘being vegetarian’) may become a marker of *social identity* in one context (amongst vegetarians), and of *personal identity* in another context (amongst non-vegetarians).

In its original formulation, self-categorization theory proposed that *social identities* tend to be activated or “‘switched on’” (Turner et al., 1987, p. 44) at the expense of *personal identity*, since “there tends to be an inverse relationship between the salience of the personal and social levels of self-categorization” (Turner et al., 1987, p. 49).^7^ Huddy (2001) argues that *social identity* and *personal identity* are not aspects of identity that can be easily teased apart from each other. They are often entangled in complex ways, which might partly explain why there can be varying degrees of strength of *social identity* (Huddy, 2001, p. 145). She suggests that individuals who categorize themselves as members of the same group can endorse the same *social identity* to different degrees because their *personal identities* will also be involved to different extents. In light of this plausible suggestion, it seems better to drop the talk of “levels” of self-representation, with its connotations of discontinuity and hierarchical organisation, and take instead *personal* and *social identity* as two endpoints in an “identity continuum” (Oakes, 2002, p. 819; Turner et al., 1994, p. 456).

The notion of *social identity* resonates well with the features of we-identities identified in the previous section.^8^ First, *social identities* are identities that individuals have qua members of social groups. Moreover, according to self-categorization theory, *social identity* is not merely an external categorization but rather a psychological property that is “phenomenologically real” (Hogg & Abrams, 1990, p. 7). What is at stake in *social identity* is “group membership as a psychological […] state, […] the subjective sense of togetherness, we-ness, or belongingness” (Turner, 1982, p. 16). Furthermore, for any given categorization to become a *social identity* it has to be internalized, it has to be “accepted as self-defining” (Oakes, 2002, p. 812), which matches well with the *Endorsement Condition* mentioned above. Although, in some formulations, self-categorization theory is described as a primarily cognitive theory (Turner, 1982), it can accommodate the point that *social identities* tend to be more or less affectively salient, and that there is an “emotional significance attached to […] membership” (Tajfel, 1981, p. 255). The *prima facie* overlap between the notions of *social identity* and we-identity makes natural to consider the question of whether the account that self-categorization theory provides of *social identities* can be applied to we-identities tout court.

The important question becomes, of course, how self-categorization theory explains the elicitation of *social identities*. The theory answers this question by positing a process of “de-personalization” as the one through which “people come to perceive themselves more as the interchangeable exemplars of a social category than as unique personalities defined by their individual differences from others” (Turner et al., 1987, p. 50). The proposal that “depersonalization of self-perception is the basic process underlying group phenomena” is at the core of self-categorization theory (Turner et al., 1987, p. 50; Hornsey, 2008, p. 208). Importantly, the relevant notion of *depersonalization* shouldn’t be conflated with any psychopathological symptom or disorder.^9^*Depersonalization* is neither a loss of individual identity, nor a dehumanizing apprehension (Hogg, 2006, p. 118), but rather a “*change* from the personal to the social level of identity” (Turner et al., 1987, pp. 51, 204).

Such a change is not supposed to be just a self-related process. It also has repercussions for how others are apprehended: “[t]argets are no longer represented as unique individuals but, rather, as embodiments of the relevant prototype” (Hogg & Terry 2000, 123). Consequently, the activation of a *social identity*, via the process of *depersonalization*, entails a certain transformation of the identity of self and others, although—as suggested above—it seems best not to think of such transformation as a shift between different levels, but rather as a graded modification along a continuum.

In sum, according to the theory of self-categorization, being ‘one of us’ is a matter of apprehending oneself and others as relatively uniform and homogeneous. Such homogeneity is not a mere abstraction. It is experienced from within, and it can be further specified in terms of the perceived interchangeability amongst group members with respect to relevant attributes. Perceived interchangeability, in turn, is grounded in an apprehension of oneself and others as more or less optimal exemplifications of a relevant in-group prototype, rather than as unique and idiosyncratic individuals.

Similar ideas can be found in classical and contemporary phenomenological contributions on the we. Although social homogeneity and interchangeability are famously emphasized in Heidegger’s description of “the One [*das Man*]” (Heidegger, 2001, p. 164; see also Gurwitsch, 1979, p. 130), consider Gerda Walther’s less well-known distinction between the “public or social person” and the “private person” (Walther, 1923, p. 105; see also Stein, 2000, pp. 134–135). The “public person” encompasses for Walther experiences and actions in which “the subject no longer lives as “itself,” […] but rather as a “representative” of the community, “in its name,” it considers itself only as a point of actualization and passage of that other, here the community, in whose name it experiences, as its “mouthpiece”” (Walther, 1923, p. 104. My translation).^10^ Walther’s examples suggest that she thinks of the public person in terms of relatively stable and recognized social roles (such as being a policeman or being a judge), which is not exactly how self-categorization theory conceptualizes the highly labile *social identities* (see, however, Hogg, 2001, p. 132). Nonetheless, Walther provides an early conceptualization of the distinction—and possible tensions (1923, p. 105)—between two aspects of self-identity, one linked to the uniqueness of the individual and the other to the individual’s perceived belongingness to social groups—which is, after all, a core tenet of the theory of self-categorization. In different ways, both Walther’s notion of the “public person” and Heidegger’s notion of “the One” suggest an understanding of the we as a form of being together in the public domain, be it *in the name of* a community that one is part of or amongst others “from whom, for the most part, one does *not* distinguish oneself” (Heidegger, 2001, p. 154).

According to one prominent contemporary phenomenological approach informed by self-categorization theory, the adoption of a first-person plural perspective requires a downplaying of interpersonal differences and the elicitation of some degree of “social uniformity” (Zahavi, 2019, p. 256). Although such uniformity is ultimately anchored in each experiencing subject, one guiding idea of this approach is that via socially mediated forms of self-apprehension, the radical difference between self and other can be downplayed, thereby allowing for some integration and unification. By taking and internalizing another’s perspective on oneself, one can undergo a process of self-transformation (2019, p. 255)—also referred to as “self-alienation” (cf. Husserl, 1973c, p. 634)—, apprehend oneself as like the other and see oneself as “one of us” (Zahavi, 2014, p. 247). It should be noted that self-categorization theory was developed as an alternative to social-psychological approaches that foreground the role of interpersonal interaction and attraction between individuals in the elicitation of group cohesion (Turner et al., 1987, ch. 5). Nonetheless, there would be a convergence between *depersonalization* and the Husserlian idea of self-alienation, insofar as both are taken to elicit some degree of social uniformity (Zahavi, 2019, p. 256).^11^

## We-identities beyond self-categorization theory

The main issue to be discussed in the rest of this paper concerns the scope of applicability of the approach to we-identities offered by self-categorization theory. Turner and colleagues are quite unequivocal in their endorsement of the claim that “depersonalization of self-perception is the basic process underlying group phenomena” (1987, p. 50, 1994, pp. 455, 460). Since self-categorization typically focuses on large-scale groups—such as cultural groups, nations, etc.—, in which members don’t have to know one another, one might think that the theory is not intended to be applicable to small-scale groups, such as, say, groups of friends or close relatives.

It is quite tempting indeed to rely on a rough-and-ready distinction between small-scale and large-scale groups, based perhaps on paradigmatic examples of each category. This is not, however, an assumption taken for granted by Turner and co-authors, who explicitly seek to circumvent the difficulty of how to provide a rigorous distinction between small and large groups (1987, pp. 103, 99) by proposing that “a collection of individuals- *and there is no theoretical restriction on number* - becomes a group to the extent that it acts in terms of a shared ingroup category” (Turner et al., 1987, p. 103 my emphasis).^12^ As Hogg clarifies, “[t]he point here is not that group size has no effect on the group, but that the definition of what constitutes a psychological group should not depend on group size” (1992, p. 66).^13^ These bold statements are plausibly related to the ambition of self-categorization theory to provide an answer to the general question of “how individuals are able to act as a group at all” (Turner et al., 1987, p. 42).

Independently of the merits that the approach provided by self-categorization theory has for the investigation of *some* we-identities, I will argue that there is a range of familiar and significant we-identities that don’t fall under the category of *social identities*. These are the we-identities that arise in the context of close personal relationships and intimacy groups, paradigmatically exemplified by long-standing romantic partnerships and friendships. On behalf of self-categorization theory, one might of course point out here that close relationships like these should be located in the domain of *personal identity*, and not of *social identity* (Hogg, 2001, p. 133). If so, it shouldn’t come as a surprise that the process of *depersonalization* is not applicable to them. This reply might be convincing within the limits of the self-categorization theory, at least to some extent.^14^

But it is much less convincing from the perspective of an overarching investigation of the we, such as the one pursued in some strands of contemporary phenomenology. Recall that contemporary phenomenological research on the we has what I called in Section 3 a broad scope. It is framed as an investigation of *the* we, and not of *a* (particular type of) we. However, in light of this broad scope, a certain discrepancy emerges. For there doesn’t seem to be any principled reason to prioritize we-identities characterized by perceived homogeneity, interchangeability, and prototypicality over we-identities that presumably lack these features. Quite to the contrary, given the central role that close relationships play in the process of socialization throughout the life-span and their documented importance for human flourishing and well-being (e.g. Dunbar, 2018), one might think that things are the other way around. Relatedly, there is no principled reason to locate the we only under the heading of *social identities*, and thereby disregard the way in which the first-person plural perspective is related to aspects of *personal identity*. In discussing close relationships and their connection with the first-person plural perspective, I aim to redress the imbalance generated by a too narrow focus on only one subdomain of we-identities.^15^

Close relationships haven’t figured prominently in discussions about the we, although it is not uncommon to find examples of collective intentionality that involve friends (e.g. Kutz, 2000, p. 7), romantic partners (e.g. Tollefsen, 2015, p. 47), and close relatives (e.g. Gilbert, 2013, p. 78; Zahavi, 2014, p. 243). The situation is different in moral philosophy, where close relationships have played a central role in discussions about associative duties and the “puzzle of partiality”, i.e. how to reconcile the partial or preferential treatments of intimates with the impartialist demands of morality (see, e.g. Keller, 2013). Gathering ideas from the literature in moral philosophy, I will extract a characterization of close relationships that I will put into use in an argument to the effect that the *depersonalization* process—with its homogenizing and levelling-down character— is not necessary for the elicitation of a we-identity.

What is a close personal relationship? To start with, it seems appropriate to distinguish between the properties of standing in a relation *to* another person and having a relationship *with* him or her (Scheffler, 2010, p. 59). While two persons may stand in some relation to each another because of sharing a common feature (such as having the same hair colour), the requirements that have to be in place for them to have a relationship *with* one another are arguably more stringent. A close relationship (involving, at a minimum, two persons) can be characterized in terms of the following features. In the first place, it is marked by a *robust concern* to promote another person’s interests and well-being.^16^ This is part of the differential treatment that comes with being in a close relationship with someone. Robustness has at least two aspects. First and foremost, since they build on historical patterns of interaction between particular individuals, close relationships don’t persist across changes of membership: “[r]elationships are individuated by the identities of their participants; they cannot survive substitution of their participants” (Kolodny, 2003, p. 148). Robustness also means that the concern at stake should be sufficiently long and motivationally strong (cf. Brogaard, 2021, p. 38).^17^ Secondly, a close relationship is *not instrumental*, in the sense that the robust concern one has for another is not part of a transactional interaction. It happens for the other’s sake, and not primarily as a medium or instrument to achieve something else.

In the third place, such robust and non-instrumental concern is typically *reciprocal*, in that it involves patterns of interlocking attitudes. This distinguishes the closeness of a friendship or a romantic partnership—it seems hard to be someone’s friend or partner without the other person being one’s friend or partner (cf. Alfano, 2016, p. 187)—from possible cases of unilateral robust concern for someone else.^18^ Reciprocity also opens the way for building the bonds of mutual trust. Fourthly, close relationships involve *emotional vulnerabilities* to one another, in the sense that how the other fares has an impact on how one fares affectively (Scheffler, 2022, p. 3). That being said, reciprocal robust concern with emotional vulnerabilities might be present in the relationship of, suppose, two psychoanalysts who analyse each other (cf. Reiman, 1976, p. 33). Yet, the more is their relationship only professional, the more it seems plausible to assume that the concern for each other might come to an end when the therapy finishes. For this reason, it seems relevant to highlight a fifth feature of close relationships, their *open-endedness*. Such relationships extend “over the course of an indefinite period (at the limit, over the course of a life)” (Westlund, 2008, p. 558). Finally, these different features must be common knowledge for the involved persons (Alfano, 2016, p. 188; Brogaard, 2021, p. 39).^19^

There surely is some degree of stipulation in how to draw the boundary between close relationships and other varieties of social relationships. The limit between close and not-so-close relationships is admittedly fuzzy, since closeness is, of course, a matter of degree. My claim is not that the just sketched characterization can find universal approval, but rather that it is sufficient to identify a central and familiar range of cases. This would explain why similar characterizations have gotten traction in discussions in moral philosophy. Note, also, that the provided characterization, which builds on the evaluative notion of concern, focuses on what one might call the normative profile of close relationships. On a broader picture, this normative profile would have to be supplemented with the cognitive profile of close relationships, focused on the socio-cognitive processes that underpin their establishment and maintenance (more on this in Section 5).

Importantly, on the present view, close relationships don’t have to be restricted to dyadic configurations. Thus, for example, a group of three close friends can be categorized as having a close relationship along the lines suggested above. Suppose that Peter, Penny, and Paula are close friends. In light of the above, this means that they have a robust, reciprocal, and open-ended concern for one another, which involves patterns of emotional vulnerabilities for each other. Moreover, apart from fitting the just provided characterization of close relationships, we may suppose that they each have a we-identity, as characterized in Section 2. This would mean that they are members of the social group that they constitute qua friends. Although they are not aware of that membership constantly, they are aware of it occasionally. Their identity as friends is not imposed from without, but is rather something that they each endorse. Such endorsement is affectively salient, not only when things go well, but also if, say, they have had a minor fight.

The important point for what follows is that close relationships involve patterns of socio-cognitive and affective interdependence between individuals, who tend to experience themselves as ‘one of us’, and yet tend to grasp themselves and one another as unique and non-substitutable. To put it differently, being ‘one of us’ in the context of a close relationship doesn’t seem to be a matter of apprehending oneself and others as relatively homogeneous and interchangeable. So there seems to be a *prima facie* tension between the feature of non-substitutability of membership, characteristic of close relationships, and the process of *depersonalization* that self-categorization theory posits at the origin of *social identity*. 20 To relate to someone as an intimate or close other is not to relate to him or her—first and foremost at least—as an instance of a relevant in-group prototype. Were one to do so, it would be quite reasonable to question whether the relationship was close in the first place. However, according to self-categorization theory and contemporary phenomenological work on the we fuelled by it, perceived social uniformity and prototypicality are critical for the elicitation of a we-identity.

In order to see that the above-mentioned tension is not merely apparent, I should clarify that the point under dispute is not that close others may grasp themselves and others as similar in some respects. In one way, that is trivially true, since close others will have some features in common. But the mere presence of interpersonal similarities wouldn’t suffice to vindicate the applicability of self-categorization theory to close relationships. The issue at stake doesn’t concern the presence of interpersonal similarities, but the psychological mechanism(s) behind social cohesion and integration. Importantly, that the *depersonalization* process is not applicable here need not mean that close others may not eventually understand one another in terms of suitable prototypes. Apart from culturally determined prototypes (e.g. the set of attributes associated with being a ‘good friend’), one might also think of ad hoc, group-specific prototypes,^21^ for example in contexts where there is some in-group – out-group demarcation.

To exemplify, suppose that the three friends are having a picnic in a park. Suddenly, a stranger with a harassing attitude approaches them. Peter asks the stranger, *on behalf of* the group of friends, to leave them alone. In that situation, it seems plausible to assume that Peter behaves under (at least) one group-specific prototype, tied to the particular group of friends that he is part of. One should also make room for the idea that *social identities* are typically enabling conditions for the formation of close relationships, insofar as an appreciation of interpersonal differences and a recognition of the uniqueness of another tend to happen against a background of recognized similarities (Hogg, 2001, p. 134).

But it would be quite different to claim that the integration of a group of friends depends as such on a tendency towards grasping each other in a prototypical and depersonalized manner, or that three friends could not act as a group at all in the absence of *depersonalization*. Such a view would not only clash with recognizing that close others evaluate one another as non-substitutable. It would also be hard to reconcile with research in personality and social psychology that has foregrounded the notion of “we-ness” in the investigation of close relationships. “We-ness” refers to the cognitive, emotional, and behavioural integration that tends to happen between intimates (Topcu-Uzer et al., 2020; Reid et al., 2006). For example, research on the phenomenon of couple resilience indicates that committed partners tend to feel and act “as a we”, when facing challenge and adversity derived from the serious illness (e.g. cancer) of one of the partners (Skerrett & Fergus, 2015; Ahmad et al., 2017). This research highlights that the kind of “we-ness” at stake here is not about a homogenization of identities, but rather of integrating with one another while recognizing each other as different. As Skerrett and Fergus put it, “the more individuated or differentiated the ‘I’ is, the more flexible and adaptive the ‘we’ will be” (Skerrett & Fergus, 2015, p. 25). In this sense, we-ness involves an appreciation of complementarity and difference, since each party contributes to the relationship in a different way: “it is key for each partner to realize that each other’s differences also contribute to the we-ness” (Reid et al., 2006, p. 247).

## Personalized we-identities

The idea that the we-identities characteristic of close relationships are not enabled by a process of *depersonalization* might not sound as particularly controversial. Spelling it out in some detail seems important, though, in light of the entrenched tendency to think of the we or first-person plural in terms of social homogeneity and uniformity. In the context of close relationships, we-ness (which one can take as a proxy for a we-identity) arises and is consolidated via processes of increasing ‘personalization’, through which individuals develop patterns of interdependence and integration with significant others.

Such processes have been investigated in personality and social psychology for quite some time. In spite of their differences, models such as the self-expansion theory (Aron et al., 1991, 2013), attachment theory (Ainsworth, 1989; Mikulincer & Shaver, 2016), and interdependence theory (Agnew et al., 1998) concur in focusing on the role of various ‘personalizing’ psychological processes that are taken to be responsible for interpersonal integration. Yet, much like self-categorization theory, the underlying picture of social cognition that these models operate with is one according to which interpersonal integration with significant others should be ultimately located in the domain of internal representations (see, e.g. Mikulincer & Shaver, 2016, p. 67; Agnew et al., 1998, p. 952).

A somewhat related tendency to construe personalizing processes as internal or self-directed can be discerned in approaches that identify a crucial feature of close relationships in the internalization of another’s perspective on oneself. It is hard to disagree with Cocking and Matthews when they comment that “through extensive shared experience, one comes to see aspects of the world (and of oneself) through the eyes of one’s friend” (Cocking & Matthews, 2000, p. 223). This is a familiar feature of close friendships and, more generally, close relationships. By seeing themselves and the world ‘through’ the eyes of the other, close others can modulate their interactions and thereby prevent that a too strong assertion of their individuality might erode the cohesion of their relationship.^22^ But should we also say that ‘seeing oneself through the eyes of another’ is a necessary condition for interpersonal integration in the context of close relationships? The notion of “self-alienation”, referenced in Section 3, could be interpreted as gesturing towards a positive answer to this question.^23^ Recall the guiding idea: by ‘seeing oneself through the eyes of another’ the distance between self and other can be decreased and one can come to see oneself as ‘one of us’ (Zahavi, 2015a, p. 94).

Now, I take it as uncontroversial that ‘seeing oneself through the eyes of another’ is a metaphorical expression, since it is not actually possible for someone to occupy another’s first-person singular perspective and be experientially directed to something through that perspective. Moreover, there seems to be conceptual space to interpret the notion of ‘seeing’ in a broad sense. True, one can see oneself as, suppose, appropriately dressed for a party by perceiving the approving glance of one’s partner before leaving for the party. But ‘seeing’ can also be interpreted as covering other forms of appraisal and evaluation (such that, for example, one can see oneself through the eyes of a friend by, suppose, reading a birthday card in which he tells about the positive character traits that he cherishes in oneself). In any case, the important point is that what the metaphor of ‘seeing oneself through the eyes of another’ actually boils down to is a process of taking and internalizing someone else’s perspective on oneself. Such form of seeing requires that “I experience and internalize the other’s perspective on myself” and that “I take over the apprehension that others have of me” (Zahavi, 2019, p. 255, 2015a, pp. 94, 98). This is a process of (at least) second-order perspective-taking—also referred to as “a type of reflection that is intersubjectively mediated” or “iterative empathy” (Zahavi, 2005, p. 94, 2014, p. 236)—, since it amounts to taking someone else’s perspective *on one’s own perspective*.

There is little doubt that perspective-taking can happen in close relationships, provided that the involved parties have the required capabilities. For example, it seems quite commonplace for close others to entertain thoughts about the other’s thoughts about themselves (e.g. Vorauer, 2012, p. 264). But is a form of higher-order perspective-taking a necessary condition for interpersonal integration and personalized we-identities? I believe that there is a more basic story to be told about how patterns of interpersonal integration in close relationships lead to the development of we-identities.^24^ Ultimately, this more basic story invites to abandon the assumption that interpersonal integration happens primarily *in foro interno*, by means of perspective-taking and the internalization of another’s perspective on oneself. It suggests instead that interpersonal integration involves the development of *relational identities*, arising from pre-reflective and affective processes through which the identities of close others become increasingly intertwined and the boundaries between them increasingly permeable.^25^

Perhaps one of the most significant indications of such permeability comes from consideration of the phenomenon of grief following the death of a long-standing and beloved life partner. As Thomas Fuchs points out, “[l]ike hardly any other psychic phenomenon, grief discloses the fact that as human beings we are fundamentally related to, and in need of others, that indeed our self is permeable and open to them” (2018, p. 48). Many first-person reports of profound grief disclose not only the outstanding affective significance that the deceased had for the bereaved person. They also indicate the affective salience attached to being ‘one of us’, since part of what is lost—or radically reconfigured (see Cholbi, 2019)—is the relationship with the other and the shared sense of an identity with him or her. This becomes particularly clear in grief reports that are partly articulated with the first-person *plural* pronoun.^26^ Moreover, a recurrent theme in first-person reports of grief is its self-involving character, indicated by the experience of a “contraction” or “partial loss of self”, often compared with an amputation (Fuchs, 2018, pp. 48, 46; Ratcliffe, 2023, pp. 56–73). For my present purposes, what matters is that grief testifies for the affective salience of the we-identities that arise in the context of close relationships, and for the extent to which patterns of interpersonal integration in such relationships put pressure on a neat demarcation between the identities of intimates.

My suggestion is that to properly account for the permeable boundaries between self and other in the context close relationships and for how such permeability is tied to the development of personalized we-identities we have to move beyond the approaches to interpersonal integration mentioned above. Such approaches either locate interpersonal integration in the domain of internal representations or else take it to be enabled by a form of reflective or higher-order perspective-taking that enriches and socially modulates what are, at bottom, self-standing and self-reliant individual identities. In the remainder of this section, I would like to sketch an alternative, externalist approach to interpersonal integration in close relationships, by bringing together resources from classical phenomenology and recent discussions about the socially extended mind. Consider the following Husserlian description of marriage and friendship:

Two human beings who build a life unity, not two lives next to each other, but two human beings, two persons, who each live his or her life and nonetheless participate in the life of the other, a co-living, a living on one’s own that is united with the other’s life, encompassing it and being encompassed by it. For the ego the other is not only someone [*ein Jemand*] who is represented in an indeterminate way as a subject of consciousness […]. The whole life of the other “belongs” also to my life, and mine to his or hers. The principle of this most intimate unity is to be determined, this most intimate unity of the two-ness [*Zweieinigkeit*] ist to be described in more detail. (Husserl, 1973b, p. 219. My translation)

Husserl suggests that the “intimate unity” of the “two-ness” involves both a tight integration and a preservation of difference. Unsurprisingly, such kind of unity goes beyond two persons who merely live their lives in parallel (“next to each other”). It refers rather to a wide-ranging co-participation in each other’s life, which, however, doesn’t erase the fact that each one lives his or her own life.^27^ One might worry that the Husserlian description is less about actual relationships of marriage and friendship, and more about a normative and culture-specific ideal that (some) people might have about them. One way to go about this worry is to recognize that interpersonal integration in close relationships, like marriage and friendship, is indeed strongly shaped by cultural norms, and yet point out that this doesn’t mean that the description doesn’t capture an occurring social phenomenon (which, of course, need not be universal). Although Husserl doesn’t elaborate much on the structure of the *Zweieinigkeit*, I suggest that discussions about the socially extended mind help to shed light on it. My discussion of the socially extended mind in the present context is intended to suggest an alternative theoretical framework to account for the personalized we-identities characteristic of close relationships. More specifically, work on the extended mind can help us to revise too individualistic assumptions about the boundaries of personhood, which may stand on the way for a proper understanding of the we-identities of close relationships.

The idea that the boundaries of the mind are not the skin and the skull has been around for some time, and it continues to attract attention and undergo new developments (see Gallagher, 2018 for a review). One recent focus of interest moves beyond the extension of various types of mental states (such as beliefs and emotions) across an agent and its environment and homes in on the idea that a self or person may extend into its environment (Heersmink, 2020). While explorations of the notions of extended selfhood or personhood have mostly focused on extension to artifacts—such as notebooks, smart phones, etc.—less attention has been paid to the idea that the identity of a person may (occasionally) extend into the identity of *other persons*. However, in light of the criteria for extension endorsed by authors who favour a “second wave” or integrationist approach to the extended mind (e.g. Sutton, 2010; Heersmink, 2015), some interesting resemblances emerge with the Husserlian description and the characterization of close relationships provided above. For the kind of tight coupling characteristic of close relationships and hinted at by Husserl resonates well with several dimensional criteria that have been proposed for a “coupled system” to approach the level of full-blown extension—such as, e.g., the presence of individualizing, reciprocal, enduring, and reliable pathways of influence (Heersmink, 2015, pp. 583–592).

Consider the notion of individualization, which has been typically used for referring to the process through which the properties of some cognitive artifacts (such as books, laptops, etc.) are adjusted and fine-tuned in such a way that they respond to the specific needs of a particular user who is tightly coupled to them (Sterelny, 2010, p. 475; Heersmink, 2015, p. 590). Analogously, two persons can individualize each other through historical and affectively laden patterns of interaction—a process that might have its roots in early ontogenesis (Greenwood, 2015, p. 646). This doesn’t mean that they have to treat one another instrumentally, merely as a suitable resource, but rather that they become distinctively sensitive to the other’s particularities, skills, and capabilities, in such a way that they become less and less substitutable for one another. When the process of individualization is properly mutual, one might even talk of an “entrenchment” (Sterelny, 2010, p. 475) of identities. The process of entrenchment or mutual individualization is affectively charged in a distinctive way, since an individualized other is not merely one person *amongst* others, but rather one who has an outstanding affective salience.

Although individualizing pathways of influence are only one dimensional criterion of extension in recent integrationist construals of the extended mind, consideration of this criterion already suggests what the socially extended mind can bring to the investigation of interpersonal integration in the context of close relationships. Moving beyond the paradigm of a homogenizing self-apprehension enabled by social interactions, interpersonal integration in close relationships can be conceptualized as an extension of the boundaries of personhood. This extension concerns, in basic cases, two persons who mutually incorporate aspects of their particular socio-cognitive and affective profiles in the context of habitualized patterns of interaction in a certain environment. The result is a tight interdependence between agents who relate to (and rely on) one another as different and individualized, and yet, at the same time, as unified in the larger whole of their relationship.

We can suppose, moreover, that this form of social extension is dramatically disrupted when one of the involved parties dies or isn’t present anymore. In such a case, the breakdown of interpersonal integration brings to the fore the affective salience of the missing other, but also of the corresponding we-identity and the sense of being ‘one of us’. The socially extended mind allows us to take at face value characterizations of grief as a contraction of the self, for it seems plausible to assume that a contracted self upon bereavement was affectively expanded or extended towards a significant other and a (particular) shared world in the first place. To be sure, my comments on the individualization criterion shouldn’t suggest that there is strict isomorphism between dimensions of extension and the features of close relationships discussed earlier, but rather that the former can illuminate the latter.

In a recent paper, Joshua Skorburg has argued that vindicating the integrationist approach to the extended mind requires a shift away from the dominant “agent-artifact paradigm” to an “agent-agent paradigm”, exemplified by research on close relationships. He reasons that “other agents are more likely than (many) artifacts to exhibit the required kind of time sensitive, reliable, and reciprocal influence” (Skorburg, 2017, p. 472). While I fully agree with Skorburg’s point, I suggest that the converse proposal is equally worth pursuing. To better understand close relationships and, in particular, the we-identities characteristic of them, we can profitably make use of the tools offered by research on the socially extended mind, in tandem with resources from classical phenomenology.

The well-known research on “transactive memory systems”, originally proposed in the context of research on intimate groups (Wegner et al., 1991; see Barnier et al., 2018), helps to buttress this point. This research indicates that long-term and cohesive romantic couples tend to develop processes of strong cognitive interdependence, including dynamic patterns of memory encoding and retrieval, in such a way that no individual member can recall in isolation what they can jointly remember.^28^ As much as self-identity over time is partly dependent on recalling, organizing, and giving coherence to past events of one’s life (Schechtman, 1996), research on transactive memory systems indicates that those can also be socially extended processes. Via processes of social extension of their identities, close others can integrate with one another into a larger systemic whole—marked linguistically with the ‘we’ pronoun—that they sustain and to which each contributes in a different way. At the same time, it is worth underlying that these contributions may be unequally distributed in the context of close relationships, given the possibility of unfairness and exploitation in the sharing of the cognitive burdens related to transactive social memory. More generally, it is important to highlight that personalized we-identities may be tied not only to positive sentiments of togetherness and unification, but also to feelings of exposure and vulnerability to significant others.

## Concluding remarks

The approach outlined in the previous section suggests that one promising way to investigate the we-identities of close-personal relationships is by synthesizing resources from classical phenomenology with the tools offered by research on the socially extended mind. This would not only allow to fill the gap of close relationships in current phenomenological literature on the we. It would also allow us to move beyond the paradigm offered by self-categorization theory, and the assumption that the we has to be thought of in terms of social uniformity and in-group prototypicality. The reason is that socially extended identities require for their stability interpersonal heterogeneity and complementarity, which are at odds with a levelling down of individual differences. If individuals who constitute a socially extended system were to level down the differences between them, the system would most likely collapse. From this perspective, the question of how individual differences might be downplayed—such that one might come to experience oneself as ‘one of us’—turns into the quite different question of how the boundaries of persons might be redrawn when they become bounded to one another in the socially extended systems of close personal relationships.

## Footnotes

1 To mark that the notion of *social identity* is a technical term in the context of the work of Turner and his collaborators (to which I will return in the next section), I will italicize it throughout the paper.

2 Note that while this definition is very broad, it is not *too* broad, since it doesn’t allow for social groups that have as members non-human animals.

3 The following discussions draws on León & Meindl (forthcoming).

4 I leave aside here the possibility that endorsement might happen under conditions of coercion or manipulation by others.

5 Self-categorization theory construes self-representations as internal to the agent, insofar as they are elements of an “information-processing system” (Turner et al., 1987, p. 44). On the one hand, self-representations are partly triggered by perceived external cues. On the other, they have as outcomes specific patterns of behaviour. By endorsing this picture, self-categorization theory is well aligned with the classical “sandwich model of the mind” (Hurley, 2001), according to which basic cognitive processes rest on the internal processing of perceptual inputs that have their source in stimuli located in the external world. Such processing, in turn, is supposed to generate as outputs certain patterns of behaviour. One open question that would merit more consideration is how current phenomenological work on the we stands vis-à-vis the internalist credentials of self-categorization theory.

6 To underline that the notion of *personal identity* is a technical term in the context of self-categorization theory I will italicize it (as I do with *social identity*).

7 Turner and co-authors also write about a “functional antagonism” between *personal identity* and *social identity*, and they refer to a “psychological discontinuity” (Turner et al., 1987, p. 66) between those two levels of self-representation.

8 At this point of my line of argument, my aim is merely to show that there is sufficient overlap between the features of we-identities identified in Section 2, and the notion of *social identity* as discussed in the context of self-categorization theory. As already mentioned in Section 2, the notion of *social identity* has the disadvantage of presupposing that it could be meaningfully distinguished from *non-social* or *pre-social* aspects of a person’s identity. However, this presupposition is highly questionable (in fact, not even Turner and co-authors take it on board). Thanks to an anonymous reviewer for prompting me to clarify this point.

9 Turner et al. consider the disadvantages of the term, but they ultimately stick to it in lack of a better alternative (Turner et al., 1987, p. 204). To mark its technical use, I italicize it.

10 Walther takes the notion of “in the name of” from Adolf Reinach, who in his ground-breaking discussion of “social acts” (1989, pp. 158–169) considers the case of performing a social act (e.g. a promise) on behalf of, or “in the name of” someone else. The conceptual innovation introduced by Walther is to apply this element of Reinach’s analysis of social acts to the relationship between individual and community.

11 How exactly one should think of the relationship between *depersonalization* and the Husserlian notion of “self-alienation” is not a question that has received much attention in the extant literature. Even though, concerning the elicited “social uniformity”, Dan Zahavi comments that “[t]he similarity to Husserl’s proposal should be obvious” (2019, p. 256), the picture seems to get more complex in light of Turner et al.’s suggestion that in self-categorization theory “the perceiver appraises self *in relation to* others, not *from* the perspective of others” (1994, p. 460. My emphasis). In Section 5, I will consider to what extent something like the Husserlian “self-alienation” might play a role in close personal relationships, independently of my present point that both “self-alienation” and *depersonalization* are supposed to elicit social uniformity and similarity.

12 See also: “five individuals in a room have just as much become a group […] if they are behaving in terms of a large-scale category membership they share (such as race, sex, or religion) as if in terms of an emergent group whose norms and defining features are unique to that specific collection of five people (a friendship group, an experimental decision-making group, etc.). *Psychological* group formation is based on self-categorization in terms of the relevant category (whether small, ad hoc, face-to-face, short-lived experimental group or large-scale, widely dispersed, culturally produced social category)” (Turner et al., 1987, p. 103).

13 In fact, from the perspective of self-categorization theory, two individuals (or even just one) may act as a group if their identity is defined in terms of a larger collective (Hogg, 2006, p. 117).

14 In the past few decades, there has been an ongoing debate in social psychology on whether it is appropriate to supplement the taxonomy of *personal identity* and *social identity* with a third category of self-representation, which captures the self in relation to significant others. Some researchers call it “relational identity” or the “relational self” (Brewer & Gardner, 1996; Chen et al., 2011). One open question (beyond the scope of the present paper) for those sympathetic to this idea is how to understand the relationship between the “three selves” (individual, relational, and collective): are they supposed to be “partners, opponents, or strangers” (Sedikides & Brewer, 2001, p. 1)?

15 To be clear, my point is not that self-categorization theory is not useful for the investigation of the we, but rather that the unqualified reliance on that theory encourages and reinforces the discrepancy between the broad scope of phenomenological research on the we and the narrow focus on only one sub-domain of it.

16 The present characterization focuses on non-antagonistic and relatively harmonious close relationships. For this reason, I leave aside the possibility of close relationships marked by an intimate hating or a robust concern with an antagonistic character. Thanks to one anonymous reviewer for pressing this point.

17 As suggested by Brogaard, closeness doesn’t require to know each and every interest of the other person. This would be an implausible requirement. For this reason, it is useful to distinguish between a *de re* concern and a *de dicto* concern, and point out that closeness involves a *de dicto* concern to promote another person’s interests (whatever they are) (see Brogaard, 2021, p. 38). Note that my characterization differs from Brogaard’s, since I operate with the notion of concern, instead of desire.

18 Thus, on the current view, a benefactor who has a unilateral robust concern to promote the interests of person A is not in a close relationship with A. Closeness might be diminished if there is no reciprocity (one might think here of some parent-child relationships, or close relationships with disabled persons) (Brogaard, 2021, p. 39).

19 The inclusion of this common knowledge requirement (understood along the seminal contributions of Lewis (2002) and others) indicates that, on the present view, close relationships are relatively demanding phenomena, cognitively speaking.

20 The patterns of interdependence between close others are also reflected in the persistency conditions of the groups that they come to constitute. While some groups persist across changes of individual members, other groups don’t satisfy this condition. As Husserl writes: “a marriage ceases to exist when one of the spouses dies, and it is not the same marriage if the other spouse re-marries. The same applies to friendship. Things are different if a circle of friends loses members and takes in new members, and we talk about the same circle of friends” (Husserl, 1973a, p. 101. My translation).

21 See footnote 9 above.

22 Thanks to one anonymous reviewer for inviting me to elaborate on this point.

23 At the end of Section 3, I mentioned that there is a convergence between the Husserlian notion of “self-alienation” and the process of *depersonalization*, insofar as both are supposed to elicit some degree of social uniformity. The point that I am considering here is whether one could take “self-alienation” to be an enabling condition of interpersonal integration in close relationships, insofar as it allows for an interpersonally mediated consciousness of oneself.

24 Note, moreover, that it is quite dubious that higher-order perspective-taking is a sufficient condition for interpersonal integration. This is nicely illustrated by Richard Moran in his recent discussion of a scene from George Elliot’s *Middlemarch*. Moran discusses a scene in which the marriage of Dorothea Brooke and Mr. Casaubon “is disintegrating from the limited intimacy that characterized it before to a settled mutual alienation” (Moran, 2018, p. 169). In a face-to-face interaction with his wife, Mr. Casaubon seeks to withhold from Dorothea some information about his illness, while at the same time being quite sure *that she has had access to this information from a third party, and that she knows that he knows that she has had access to that information*. In this situation, even though Casaubon exercises a capacity for higher-order-perspective taking (we might say that the ill Casaubon is seeing himself through Dorothea’s evaluative eyes), the outcome of the situation is clearly not interpersonal integration, but rather, as Moran notes, “failed mutuality” (2018, p. 169).

25 I borrow the term ‘relational identities’ from Brewer and Gardner (1996), who use it (differently from me) as a specific level of (internal) self-representation (see footnote 5 above). Moreover, I should note that the notion of permeability that I use in the present context is intended to reference the fluid boundaries of interpersonal concern that intimates have for one another. As such, it shouldn’t be conflated with the idea of a fusion of streams of consciousness.

26 Consider the following examples: “I am grieving not only him, but the loss of *our life together*, past, and future. (#17)”; “Losing my husband meant losing the future I thought I had, the everyday routine that *we* had, the security I felt and the deep love that *we* shared. (#41)” (quoted in Ratcliffe, 2023, p. 159 My emphasis). My present comments on grief go rather quickly over complex issues in grief research, to which I hope to come back in future work.

27 A similar idea is expressed by Merleau-Ponty: “To love is inevitably to enter into an undivided situation with another. […] One is not what one would be without that love; the perspectives remain separate—and yet they overlap.” (Merleau-Ponty, 1964, p. 155). See also Binswanger’s description of the interplay between (dyadic) togetherness and loneliness, in the context of the “dual we-ness” of the “loving being-with-one-another”: “The larger the reality of dyadic togetherness [*Zweisamkeit*], the greater the possibility of loneliness [*Einsamkeit*], and the larger the reality of loneliness, the greater the possibility of dyadic togetherness” (Binswanger, 1993, p. 118. My translation).

28 As suggested by Tollefsen (2006, p. 145), this indicates that joint remembering in the context of transactive memory systems cannot be properly located ‘inside’ the minds of the individual agents, insofar as it emerges from their dynamic communicative interaction. This lends support to the idea that some processes of joint remembering are socially extended (and puts pressure on alternative, internalist explanations).

## References

[CR1] Agnew, C. R., Van Lange, P. A. M., Rusbult, C. E., & Langston, C. A. (1998). Cognitive interdependence: Commitment and the mental representation of close relationships. *Journal of Personality and Social Psychology,**74*(4), 939–954. 10.1037/0022-3514.74.4.939

[CR2] Ahmad, S., Fergus, K., Shatokhina, K., & Gardner, S. (2017). The closer ‘We’ are, the stronger ‘I’ am: The impact of couple identity on cancer coping self-efficacy. *Journal of Behavioral Medicine,**40*(3), 403–413. 10.1007/s10865-016-9803-1 27848061 10.1007/s10865-016-9803-1

[CR3] Ainsworth, M. S. (1989). Attachments beyond infancy. *American Psychologist,**44*(4), 709–716. 10.1037/0003-066X.44.4.709 2729745 10.1037//0003-066x.44.4.709

[CR4] Alfano, M. (2016). Friendship and the structure of trust. In A. Masala & J. Webber (Eds.), *From personality to virtue: Essays on the philosophy of character* (pp. 186–206). Oxford University Press.

[CR5] Appiah, A. (2014). *Lines of descent: W. E. B. Du Bois and the emergence of identity*. Harvard University Press.

[CR6] Aron, A., Aron, E. N., Tudor, M., & Nelson, G. (1991). Close relationships as including other in the self. *Journal of Personality and Social Psychology,**60*(2), 241–253. 10.1037/0022-3514.60.2.241

[CR7] Aron, A., Lewandowski, G. W., Mashek, D., & Aron, E. N. (2013). The Self-Expansion Model of Motivation and Cognition in Close Relationships. In J. Simpson, & L. Campbell (Eds.), *The Oxford Handbook of Close Relationships*. Oxford University Press. 10.1093/oxfordhb/9780195398694.013.0005

[CR8] Ashmore, R. D., Deaux, K., & McLaughlin-Volpe, T. (2004). An Organizing Framework for collective identity: Articulation and significance of Multidimensionality. *Psychological Bulletin,**130*(1), 80–114. 10.1037/0033-2909.130.1.80 14717651 10.1037/0033-2909.130.1.80

[CR9] Bacharach, M. (2006). In N. Gold (Ed.), *Beyond individual choice: Teams and frames in game theory. *Princeton University Press.

[CR10] Barnier, A. J., Klein, L., & Harris, C. B. (2018). Transactive Memory in Small, intimate Groups: More than the Sum of their parts. *Small Group Research,**49*(1), 62–97. 10.1177/1046496417712439

[CR11] Baumeister, R. F., & Leary, M. R. (1995). The need to belong: Desire for interpersonal attachments as a fundamental human motivation. *Psychological Bulletin,**117*(3), 497–529. 10.1037/0033-2909.117.3.497 7777651

[CR12] Binswanger, L. (1993). *Grundformen und Erkenntnis menschlichen Daseins*. Asanger.

[CR13] Brewer, M. B., & Gardner, W. (1996). Who is this we? Levels of collective identity and self representations. *Journal of Personality and Social Psychology,**71*(1), 83–91.

[CR14] Brogaard, B. (2021). Practical identity and duties of love. *Disputatio,**13*(60), 27–50. 10.2478/disp-2021-0002

[CR15] Brownlee, K. (2020). *Being sure of each other: An essay on social rights and freedoms*. Oxford University Press.

[CR16] Chen, S., Boucher, H., & Kraus, M. W. (2011). The relational self. In S. J. Schwartz, K. Luyckx, & V. L. Vignoles (Eds.), *Handbook of Identity Theory and Research* (pp. 149–175). Springer. 10.1007/978-1-4419-7988-9_7

[CR17] Cholbi, M. (2019). Regret, resilience, and the nature of grief. *Journal of Moral Philosophy,**16*(4), 486–508. 10.1163/17455243-20180015

[CR18] Cocking, D., & Matthews, S. (2000). Unreal friends. *Ethics and Information Technology,**2*(4), 223–231. 10.1023/A:1011414704851

[CR19] Dunbar, R. I. M. (2018). The anatomy of friendship. *Trends in Cognitive Sciences,**22*(1), 32–51. 10.1016/j.tics.2017.10.004 29273112 10.1016/j.tics.2017.10.004

[CR20] Epstein, B. (2019). What are social groups? Their metaphysics and how to classify them. *Synthese*, *196*(12). 10.1007/s11229-017-1387-y

[CR21] Fiske, S. T. (2014). *Social beings: Social core motives in social psychology*. John Wiley and Sons.

[CR22] Flesher Fominaya, C. (2010). Collective identity in social movements: central concepts and debates. *Sociology Compass,**4*(6), 393–404. 10.1111/j.1751-9020.2010.00287.x

[CR23] Fuchs, T. (2018). Presence in absence. The ambiguous phenomenology of grief. *Phenomenology and the Cognitive Sciences,**17*(1), 43–63. 10.1007/s11097-017-9506-2

[CR24] Gallagher, S. (2018). The extended mind: State of the question. *The Southern Journal of Philosophy,**56*(4), 421–447. 10.1111/sjp.12308

[CR25] Gallagher, S. (2020). *Action and interaction*. Oxford University Press.

[CR26] Gilbert, M. (2013). *Joint commitment: How we make the Social World*. Oxford University Press. 10.1093/acprof:oso/9780199970148.001.0001

[CR27] Greenwood, J. (2015). Is mind extended or scaffolded? Ruminations on Sterelney’s (2010) extended stomach. *Phenomenology and the Cognitive Sciences,**14*(3), 629–650. 10.1007/s11097-013-9337-8

[CR28] Gurwitsch, A. (1979). *Human encounters in the Social World*. Duquesne University Press.

[CR29] Heersmink, R. (2015). Dimensions of integration in embedded and extended cognitive systems. *Phenomenology and the Cognitive Sciences,**14*(3), 577–598. 10.1007/s11097-014-9355-1

[CR30] Heersmink, R. (2020). Varieties of the extended self. *Consciousness and Cognition,**85*, 103001. 10.1016/j.concog.2020.103001 10.1016/j.concog.2020.103001 32823060

[CR31] Heidegger, M. (2001). *Being and time*. Blackwell.

[CR32] Hogg, M. A. (1992). *The social psychology of group cohesiveness: From attraction to social identity*. Harvester-Wheatsheaf.

[CR33] Hogg, M. A. (2001). Social Identity and the Sovereignty of the Group: A psychology of belonging. In C. Sedikides, & M. B. Brewer (Eds.), *Individual self, relational self, collective self* (pp. 125–143). Psychology Press.

[CR34] Hogg, M. A. (2006). Social identity theory. In P. J. Burke (Ed.), *Contemporary social psychological theories* (pp. 111–136). Stanford Social Sciences.

[CR35] Hogg, M. A., & Abrams, D. (1990). *Social identifications. A social psychology of intergroup relations and group processes*. Routledge.

[CR36] Hogg, M. A., & Terry, D. J. (2000). Social identity and self-categorization processes in organizational contexts. *The Academy of Management Review,**25*(1), 121–140. 10.2307/259266

[CR37] Hornsey, M. J. (2008). Social Identity Theory and Self-categorization theory: A historical review. *Social and Personality Psychology Compass,**2*(1), 204–222. 10.1111/j.1751-9004.2007.00066.x

[CR38] Huddy, L. (2001). From Social to Political Identity: A critical examination of Social Identity Theory. *Political Psychology,**22*(1), 127–156. 10.1111/0162-895X.00230

[CR39] Hurley, S. (2001). Perception and action: Alternative views. *Synthese,**129*(1), 3–40. 10.1023/A:1012643006930

[CR40] Husserl, E. (1973a). *Zur Phänomenologie der Intersubjektivität I. Texte aus dem nachlass. Erster Teil. 1905–1920*. Martinus Nijhoff.

[CR41] Husserl, E. (1973b). *Zur Phänomenologie der Intersubjektivität II. Texte aus dem nachlass. Zweiter Teil. 1921-28*. Martinus Nijhoff.

[CR42] Husserl, E. (1973c). *Zur Phänomenologie der Intersubjektivität III. Texte aus dem nachlass. Dritter Teil. 1929-35*. Martinus Nijhoff.

[CR43] Keller, S. (2013). *Partiality*. Princeton University Press.

[CR44] Kolodny, N. (2003). Love as valuing a relationship. *Philosophical Review,**112*(2), 135–189. 10.1215/00318108-112-2-135

[CR45] Kutz, C. (2000). Acting together. *Philosophy and Phenomenological Research*, *61*(1). 10.2307/2653401

[CR46] León, F., & Meindl, P. (forthcoming). The stranger and the homecomer: Two cases studies for a phenomenology of belonging. In L. Dolezal & D. Petherbridge (Eds.), *Phenomenology of Belonging. *State University of New York Press.

[CR47] Lewis, D. (2002). *Convention: A philosophical study*. Blackwell.

[CR48] Lickel, B., Hamilton, D. L., & Sherman, S. J. (2001). Elements of a Lay Theory of Groups: Types of groups, relational Styles, and the perception of Group Entitativity. *Personality and Social Psychology Review,**5*(2), 129–140. 10.1207/S15327957PSPR0502_4

[CR49] Mathiesen, K. (2003). On collective identity. *ProtoSociology,**18*, 66–86. 10.5840/protosociology200318/192

[CR50] Merleau-Ponty, M. (1964). *The primacy of perception*. Northwestern University Press.

[CR51] Mikulincer, M., & Shaver, P. R. (2016). *Attachment in adulthood: Structure, dynamics, and change*. Guilford Press.

[CR52] Moran, R. (2018). *The exchange of words: Speech, testimony, and intersubjectivity*. Oxford University Press.

[CR53] Oakes, P. (2002). Psychological groups and political psychology: A response to Huddy’s ‘Critical examination of Social Identity Theory’. *Political Psychology,**23*(4), 809–824.

[CR54] Ratcliffe, M. (2023). *Grief worlds: A study of emotional experience*. The MIT Press.

[CR55] Reid, D. W., Dalton, E. J., Laderoute, K., Doell, F. K., & Nguyen, T. (2006). Therapeutically Induced Changes in Couple Identity: The Role of We-ness and Interpersonal Processing in Relationship Satisfaction. *Genetic, Social, and General Psychology Monographs*, *132*(3), 241–284. 10.3200/MONO.132.3.241-288 10.3200/mono.132.3.241-288 17970000

[CR56] Reiman, J. H. (1976). Privacy, intimacy, and personhood. *Philosophy and Public Affairs,**6*(1), 26–44. 10.4324/9781315246024-3

[CR57] Reinach, A. (1989). In K. Schuhmann, & B. Smith (Eds.), *Sämtliche Werke: Textkritische Ausgabe in 2 Bänden. Band 1*. Philosophia-Verlag.

[CR58] Rucht, D. (1995). Kollektive Identitat: Konzeptuelle Überlegungen zu einem Desiderat der Bewegungsforschung. *Forschungsjournal Neue Soziale Bewegungen,**8*(1), 9–23.

[CR59] Salice, A., & Henriksen, M. G. (2015). The disrupted we. Schizophrenia and collective intentionality. *Journal of Consciousness Studies,**22*(7–8), 145–171.

[CR60] Salice, A., & Henriksen, M. G. (2021). Disturbances of Shared Intentionality in Schizophrenia and Autism. *Frontiers in Psychiatry,**11*, 1–19. 10.3389/fpsyt.2020.570597 10.3389/fpsyt.2020.570597 PMC7902514 33643078

[CR61] Salice, A., & Miyazono, K. (2020). Being one of us. Group identification, joint actions, and collective intentionality. *Philosophical Psychology,**33*(1), 42–63. 10.1080/09515089.2019.1682132

[CR62] Schechtman, M. (1996). *The Constitution of Selves*. Cornell University Press.

[CR63] Scheffler, S. (2010). *Equality and tradition: Questions of value in moral and political theory*. Oxford University Press.

[CR64] Scheffler, S. (2022). XIV—Partiality, deference, and Engagement. *Proceedings of the Aristotelian Society,**122*(3), 319–341. 10.1093/arisoc/aoac012

[CR65] Schmid, H. B. (2005). Wir-Identität: Reflexiv und vorreflexiv. *Deutsche Zeitschrift Für Philosophie*, *53*(3). 10.1524/dzph.2005.53.3.365

[CR66] Schweikard, D., & Schmid, H. B. (2020). Collective intentionality. In *Stanford Encyclopedia of Philosophy*. https://plato.stanford.edu/entries/collective-intentionality/. Accessed 25 Apr 2023

[CR67] Sedikides, C., & Brewer, M. B. (Eds.). (2001). *Individual self, relational self, collective self*. Psychology Press.

[CR68] Simon, B., & Klandermans, B. (2001). Politicized collective identity: A social psychological analysis. *American Psychologist,**56*(4), 319–331. 10.1037/0003-066X.56.4.319 11330229

[CR69] Skerrett, K., & Fergus, K. (Eds.). (2015). *Couple resilience: Emerging perspectives*. Springer. 10.1007/978-94-017-9909-6

[CR70] Skorburg, J. A. (2017). Lessons and new directions for extended cognition from social and personality psychology. *Philosophical Psychology,**30*(4), 458–480. 10.1080/09515089.2017.1282606

[CR71] Stein, E. (2000). *Philosophy of psychology and the Humanities*. ICS Publications.

[CR72] Sterelny, K. (2010). Minds: Extended or scaffolded? *Phenomenology and the Cognitive Sciences,**9*(4), 465–481. 10.1007/s11097-010-9174-y

[CR73] Sullivan, G. B. (2014). Introduction. In G. B. Sullivan (Ed.), *Understanding collective pride and group identity: New directions in emotion theory, research and practice* (pp. 1–17). Routledge.

[CR74] Sutton, J. (2010). Exograms and Interdisciplinarity: History, the extended mind, and the civilizing process. In R. Menary (Ed.), *The extended mind* (pp. 189–225). The MIT Press.

[CR75] Taipale, J. (2019). The structure of Group Identification. *Topoi,**38*(1), 229–237. 10.1007/s11245-017-9463-y

[CR76] Tajfel, H. (Ed.). (1978). *Differentiation between social groups: Studies in the social psychology of intergroup relations*. Academic.

[CR77] Tajfel, H. (1981). *Human groups and social categories: Studies in social psychology*. Cambridge University Press.

[CR78] Tajfel, H., & Turner, J. C. (1986). The social identity theory of intergroup behavior. In W. Austin & S. Worchel (Eds.), *Psychology of intergroup relations* (pp. 7–24). Nelson-Hall Publishers.

[CR79] Tollefsen, D. (2006). From extended mind to collective mind. *Cognitive Systems Research,**7*(2–3), 140–150. 10.1016/j.cogsys.2006.01.001

[CR80] Tollefsen, D. (2015). *Groups as agents*. Polity.

[CR81] Topcu-Uzer, C., Randall, A. K., Vedes, A. M., Reid, D., & Bodenmann, G. (2020). We-ness questionnaire: Development and validation. *Journal of Couple & Relationship Therapy,**20*(3), 256–278. 10.1080/15332691.2020.1805082

[CR82] Turner, J. C. (1982). Towards a cognitive redefinition of the social group. In H. Tajfel (Ed.), *Social identity and intergroup relations* (pp. 15–40). Cambridge University Press.

[CR83] Turner, J. C., Hogg, M. A., Oakes, P. J., Reicher, S. D., & Wetherell, M. S. (1987). *Rediscovering the social group: Self-categorization theory*. Blackwell.

[CR84] Turner, J. C., Oakes, P. J., Haslam, S. A., & McGarty, C. (1994). Self and collective: Cognition and Social Context. *Personality and Social Psychology Bulletin*, *20*(5), 10.1177/0146167294205002

[CR85] Vorauer, J. D. (2012). Do you see what I see? Antecedents, consequences, and remedies for biased metacognition in close relationships. *Social metacognition* (pp. 263–281). Psychology Press.

[CR86] Walther, G. (1923). Zur Ontologie der sozialen Gemeinschaften. *Jahrbuch für Philosophie und phänomenologische Forschung,**VI*, 1–158.

[CR87] Wegner, D. M., Erber, R., & Raymond, P. (1991). Transactive memory in close relationships. *Journal of Personality and Social Psychology,**61*(6), 923–929. 10.1037/0022-3514.61.6.923 1774630 10.1037//0022-3514.61.6.923

[CR88] Westlund, A. C. (2008). The Reunion of Marriage. *The Monist,**91*(3), 558–577. 10.5840/monist2008913/430

[CR89] Young, I. M. (1990). *Justice and the politics of difference*. Princeton University Press.

[CR90] Zahavi, D. (2005). *Subjectivity and selfhood: Investigating the first-person perspective*. The MIT Press.

[CR91] Zahavi, D. (2014). *Self and other: Exploring subjectivity, empathy, and shame*. Oxford University Press.

[CR92] Zahavi, D. (2015a). You, me, and we: The sharing of emotional experiences. *Journal of Consciousness Studies,**22*(1–2), 84–101.

[CR93] Zahavi, D. (2015b). Self and other: From pure ego to co-constituted we. *Continental Philosophy Review,**48*(2), 143–160. 10.1007/s11007-015-9328-2

[CR94] Zahavi, D. (2019). Second-person engagement, self-alienation, and group-identification. *Topoi,**38*(1), 251–260. 10.1007/s11245-016-9444-6

